# Supplementary material for: Antimicrobial Activity of Gemini Surfactants with Ether Group in the Spacer Part
Source: Molecules. 2021 Sep 23;26(19):5759. doi: 10.3390/molecules26195759 (PMC8510121; doi:10.3390/molecules26195759)

SUPPLEMENTARY MATERIALS

Antimicrobial activity of gemini surfactants with ether group in the spacer part.

Bogumił Brycki, Adrianna Szulc, Iwona Kowalczyk, Anna Koziróg and Ewelina Sobolewska

Synthetic details:

4-O-4: The *N*-butyl-*N*,*N*-dimethylamine (10 g; 0.09 mol) was placed in a 100 ml round-bottom flask and bis(2-bromoethyl)ether (11.48 g; 0.045 mol) was added. Reaction was carried out without solvent, at room temperature, with stirring using a magnetic stirrer, until the reaction mixture solidified. After completion of the reaction crude product was crystallized in acetonitrile/methanol (10:1). The product was filtered and dried in an incubator (60°C) and over P_4O_{10} in the vacuum desiccator. The yield of the reaction was >98%.

6-O-6: The *N*-hexyl-*N*,*N*-dimethylamine (10 g; 0.078 mol) was placed in a 100 ml round-bottom flask and bis(2-bromoethyl)ether (8.92 g; 0.039 mol) was added. Reaction was carried out without solvent, at room temperature, with stirring using a magnetic stirrer, until the reaction mixture solidified. After completion of the reaction crude product was crystallized in acetonitrile/methanol (10:1). The product was filtered and dried in an incubator (60°C) and over P_4O_{10} in the vacuum desiccator. The yield of the reaction was >98%.

8-O-8: The *N*-octyl-*N*,*N*-dimethylamine (10 g; 0.06 mol) was placed in a 100 ml round-bottom flask and bis(2-bromoethyl)ether (7.39 g; 0.03 mol) was added. Reaction was carried out without solvent, at room temperature, with stirring using a magnetic stirrer, until the reaction mixture solidified. After completion of the reaction crude product was crystallized in acetonitrile/methanol (10:1). The product was filtered and dried in an incubator (60°C) and over P_4O_{10} in the vacuum desiccator. The yield of the reaction was >98%.

10-O-10: The *N*-decyl-*N*,*N*-dimethylamine (10 g; 0.055 mol) was placed in a 100 ml round-bottom flask and bis(2-bromoethyl)ether (6.25 g; 0.0275 mol) was added. Reaction was carried out without solvent, at room temperature, with stirring using a magnetic stirrer, until the reaction mixture solidified. After completion of the reaction crude product was crystallized in acetonitrile/methanol (10:1). The product was filtered and dried in an incubator (60°C) and over P_4O_{10} in the vacuum desiccator. The yield of the reaction was >98%.

12-O-12: The *N*-dodecyl-*N*,*N*-dimethylamine (10 g; 0.047 mol) was placed in a 100 ml round-bottom flask and bis(2-bromoethyl)ether (5.45g; 0.0235 mol) was added. Reaction was carried out without solvent, at room temperature, with stirring using a magnetic stirrer, until the reaction mixture solidified. After completion of the reaction crude product was crystallized in

acetonitrile/methanol (10:1). The product was filtered and dried in an incubator (60°C) and over P_4O_{10} in the vacuum desiccator. The yield of the reaction was >98%.

14-O-14: The *N*-tetradecyl-*N*,*N*-dimethylamine (10 g; 0.04 mol) was placed in a 100 ml round-bottom flask and bis(2-bromoethyl)ether (4.81 g; 0.02 mol) was added. Reaction was carried out without solvent, at room temperature, with stirring using a magnetic stirrer, until the reaction mixture solidified. After completion of the reaction crude product was crystallized in acetonitrile/methanol (10:1). The product was filtered and dried in an incubator (60°C) and over P_4O_{10} in the vacuum desiccator. The yield of the reaction was >98%.

16-O-16: The *N*-hexadecyl-*N*,*N*-dimethylamine (10 g; 0.037 mol) was placed in a 100 ml round-bottom flask and bis(2-bromoethyl)ether (4.30 g; 0.0185 mol) was added. Reaction was carried out without solvent, at room temperature, with stirring using a magnetic stirrer, until the reaction mixture solidified. After completion of the reaction crude product was crystallized in acetonitrile/methanol (10:1). The product was filtered and dried in an incubator (60°C) and over P_4O_{10} in the vacuum desiccator. The yield of the reaction was >98%.

18-O-18: The *N*-octadecyl-*N*,*N*-dimethylamine (10 g; 0.034 mol) was placed in a 100 ml round-bottom flask and bis(2-bromoethyl)ether (3.94 g; 0.017 mol) was added. Reaction was carried out without solvent, with stirring using a magnetic stirrer. In this case reaction mixture was heated to 30°C in water bath. The reaction was continued until it solidified (4 h). After completion of the reaction crude product was crystallized in acetonitrile/methanol (10:1). The product was filtered and dried in an incubator (60°C) and over P_4O_{10} in the vacuum desiccator. The yield of the reaction was >95%.

NMR spectra:

Compound **4-O-4**

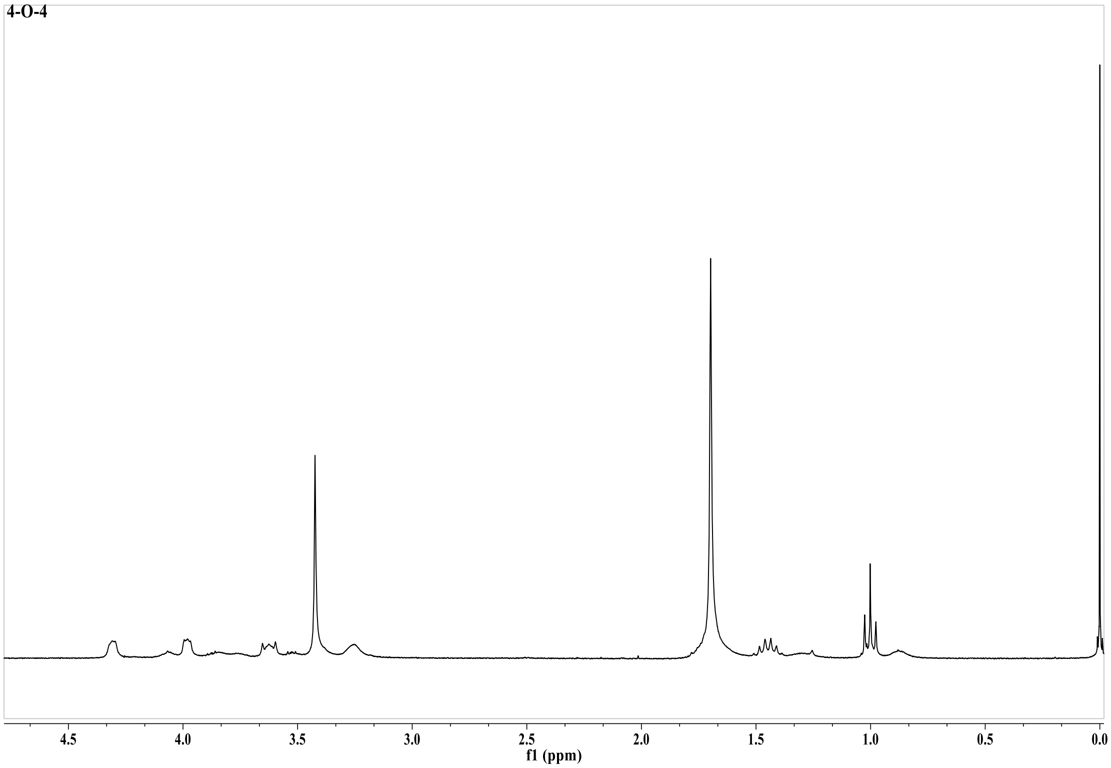

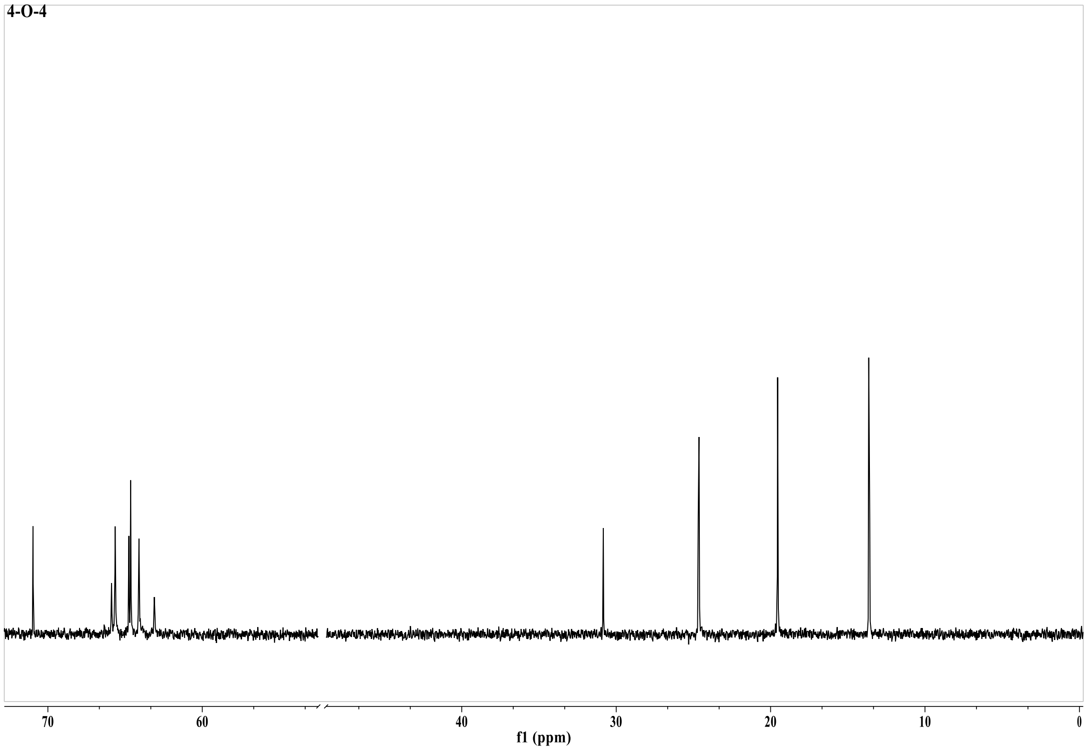

Compound **6-O-6**

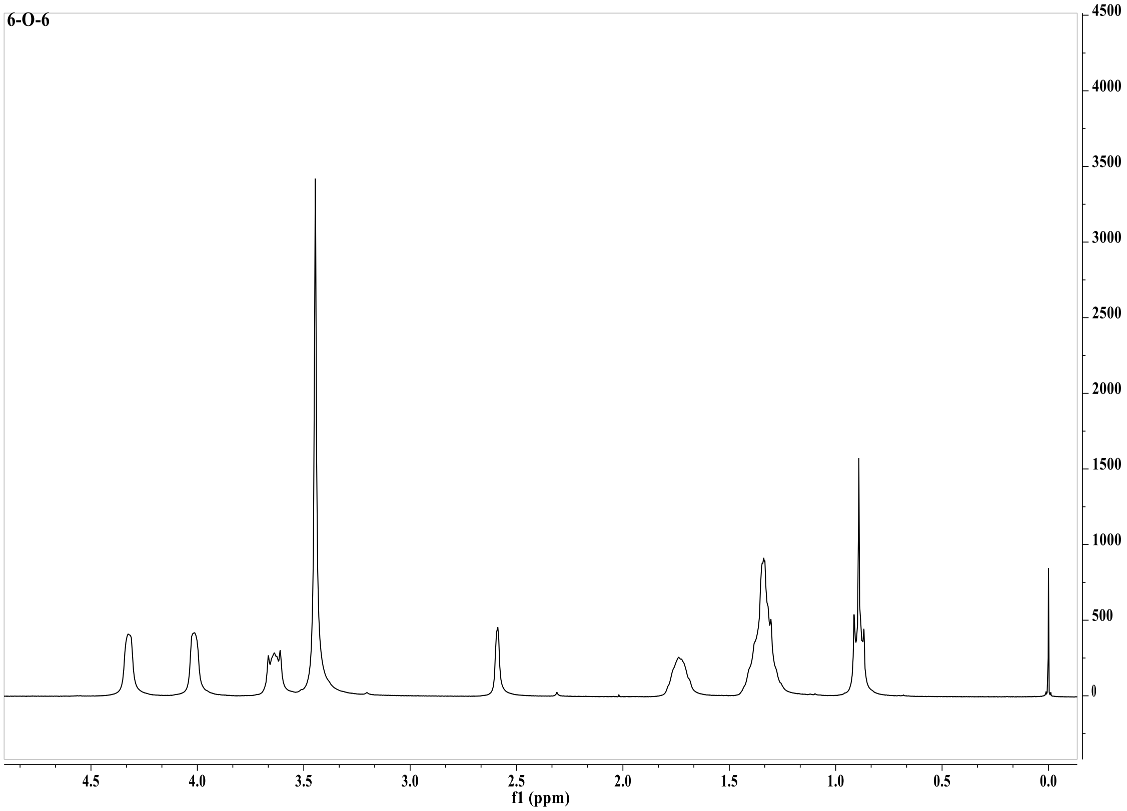

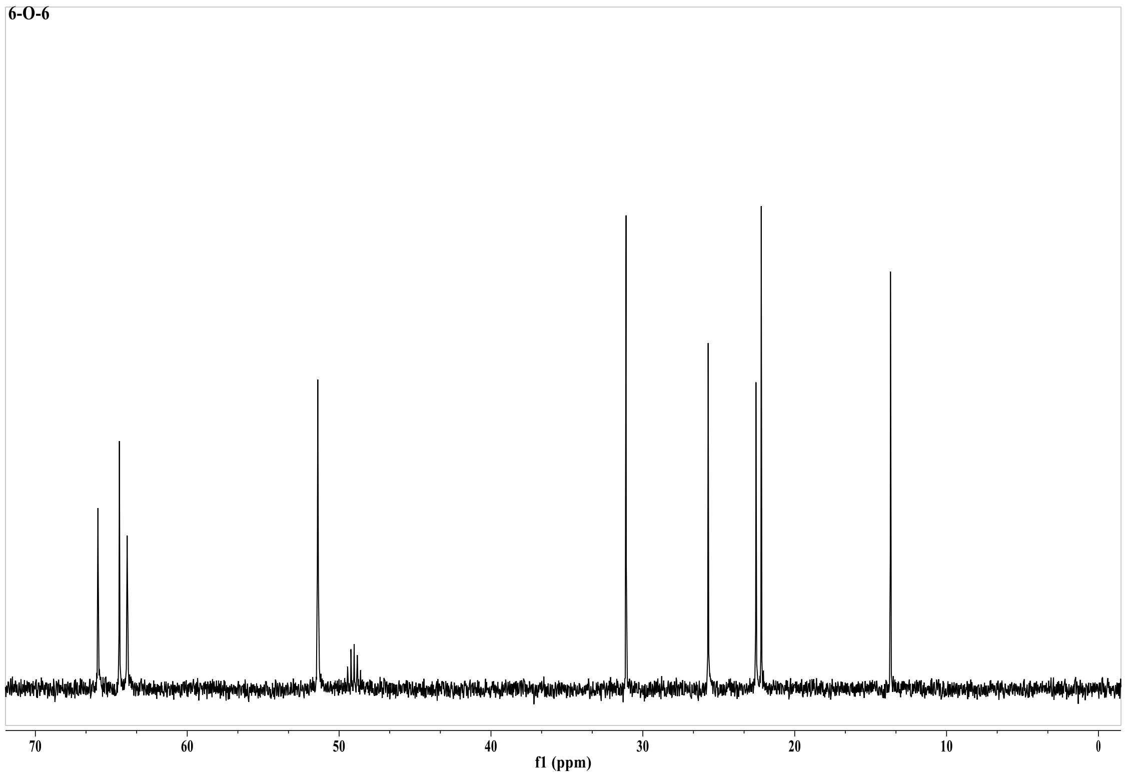

Compound **8-O-8**

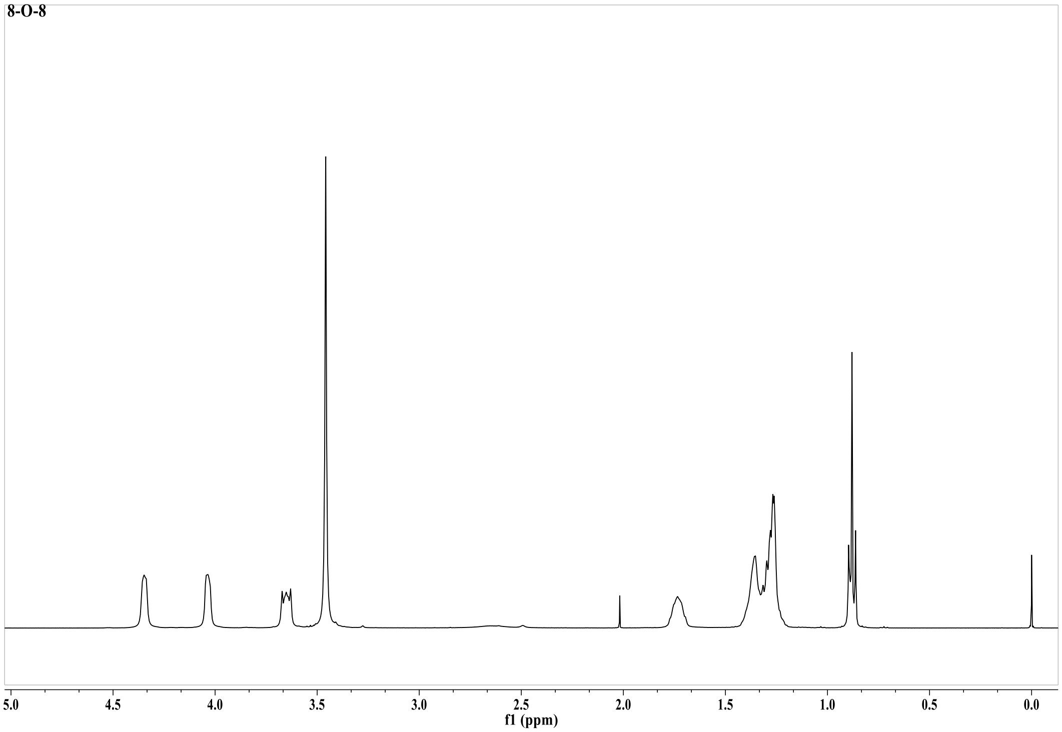

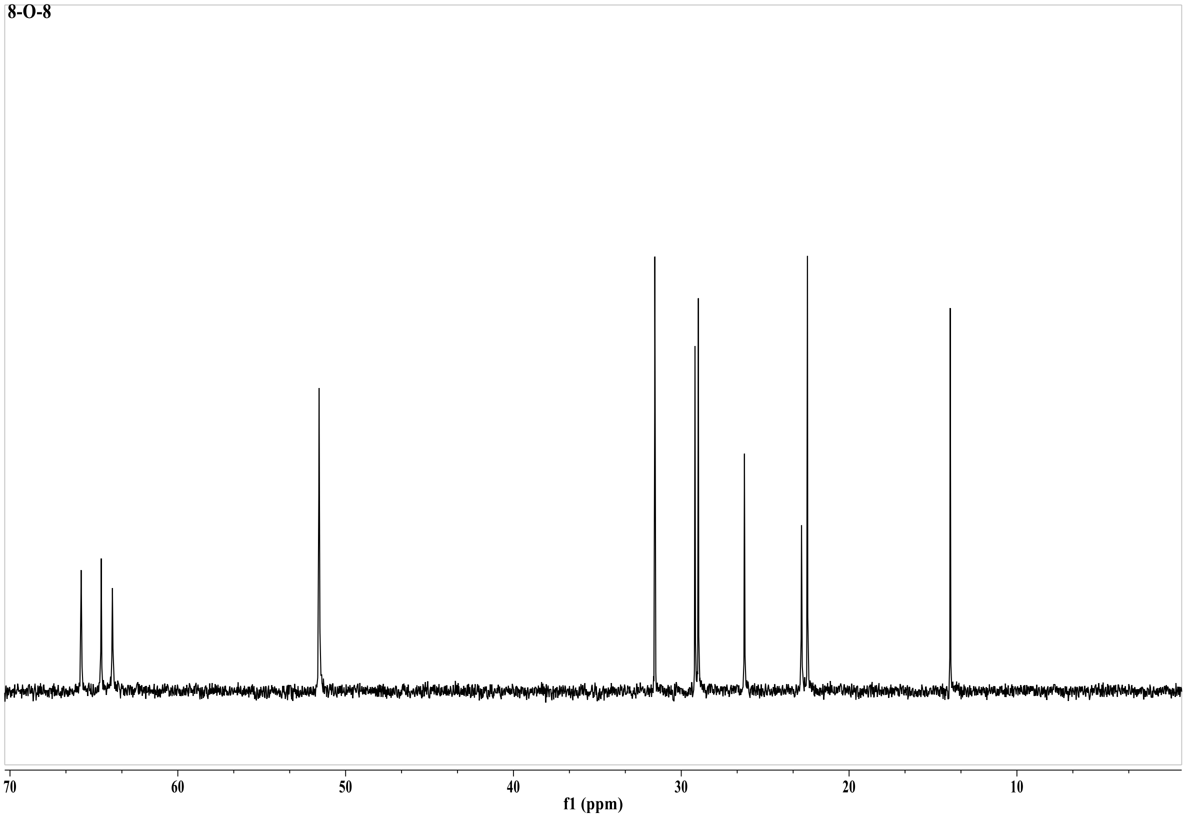

Compound **10-O-10**

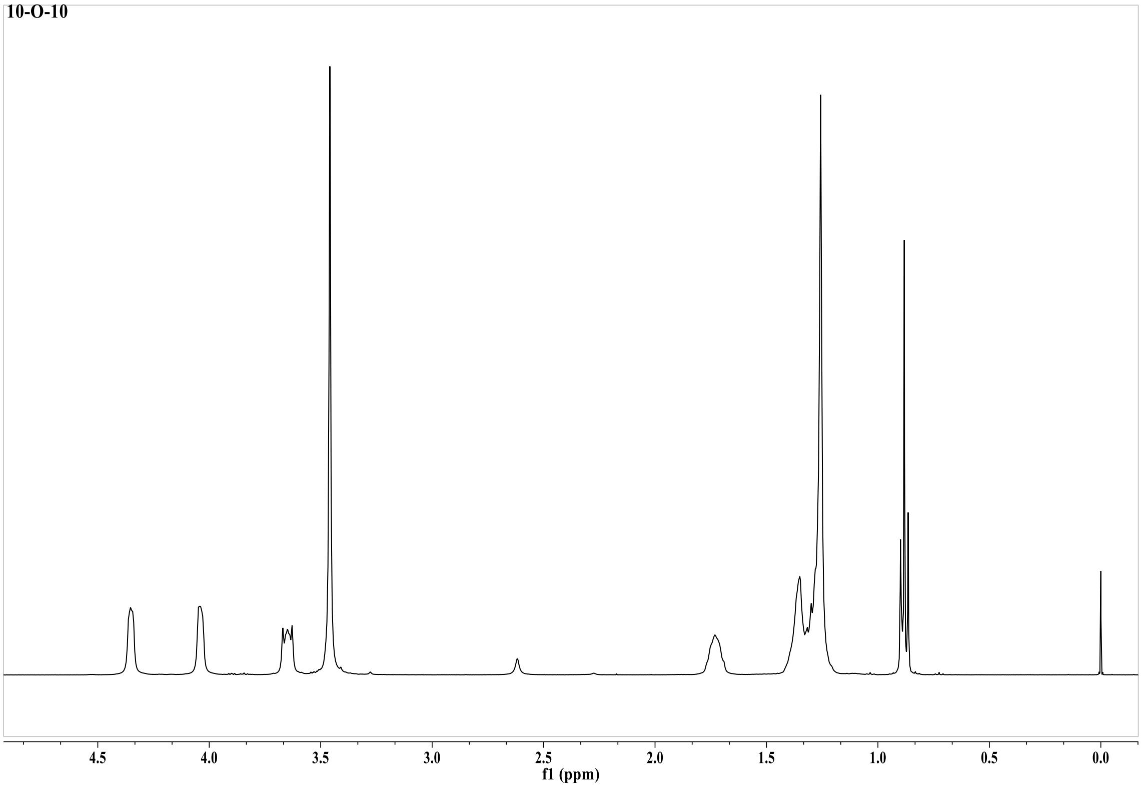

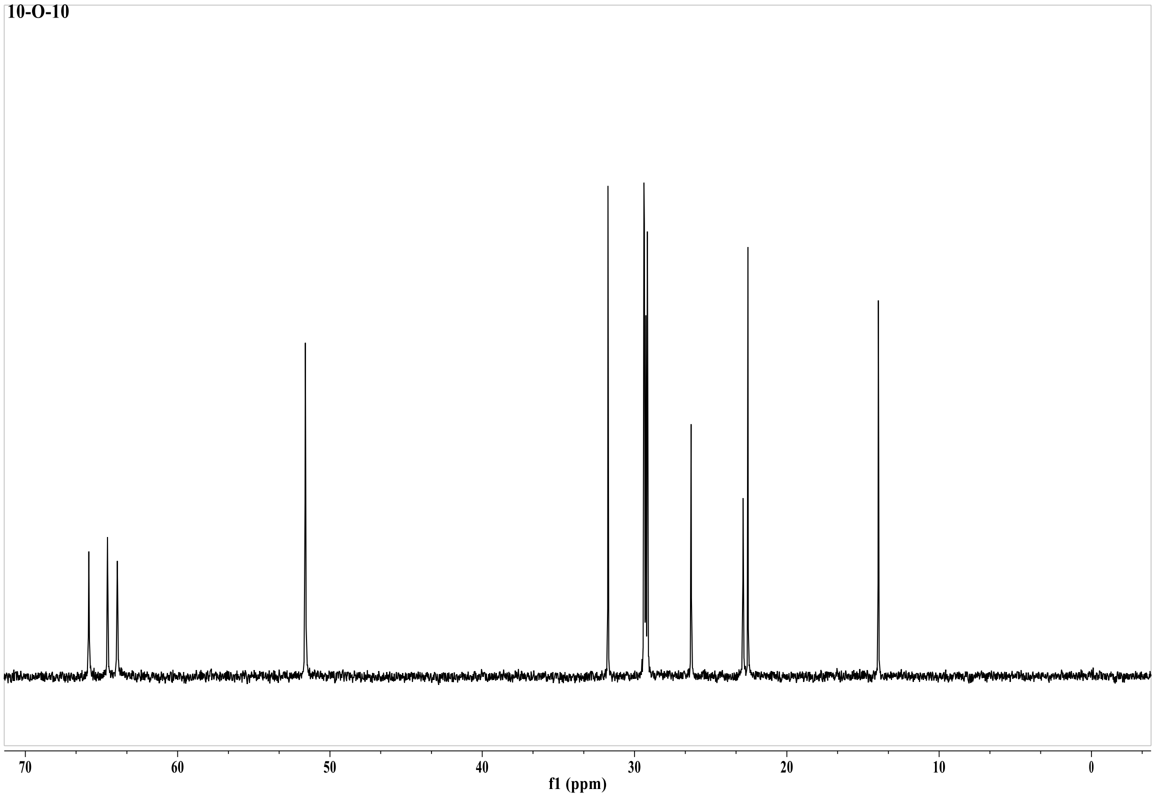

Compound **12-O-12**

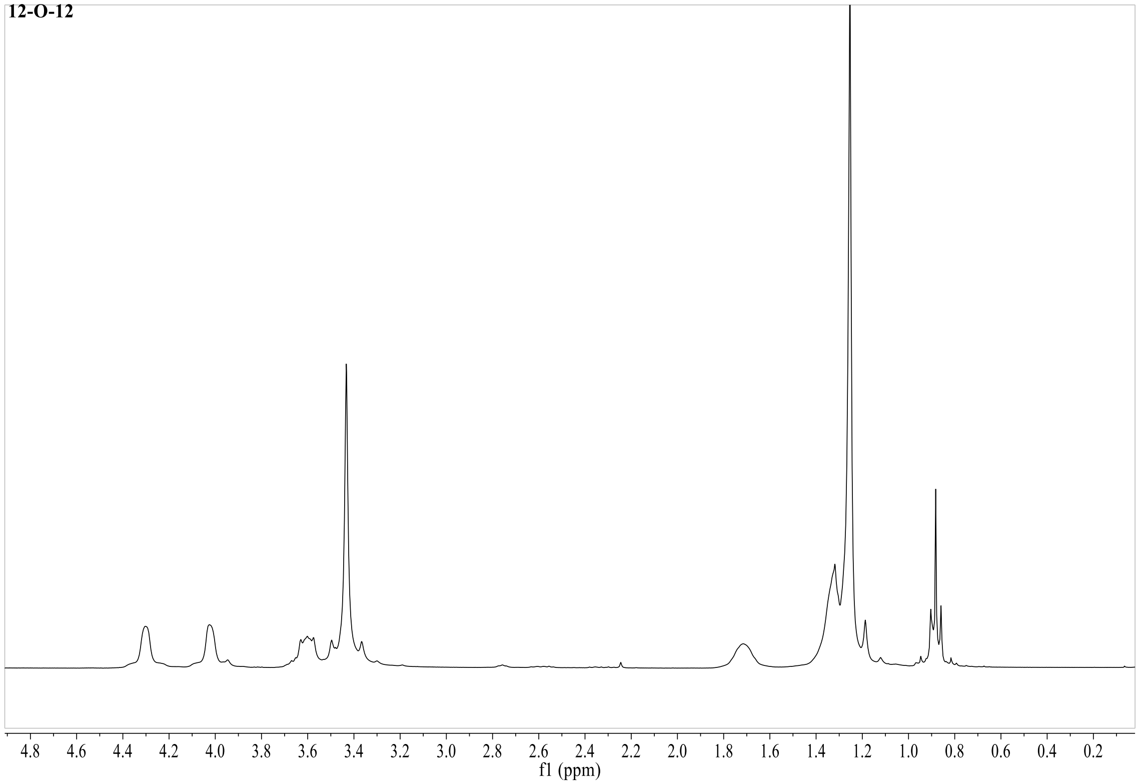

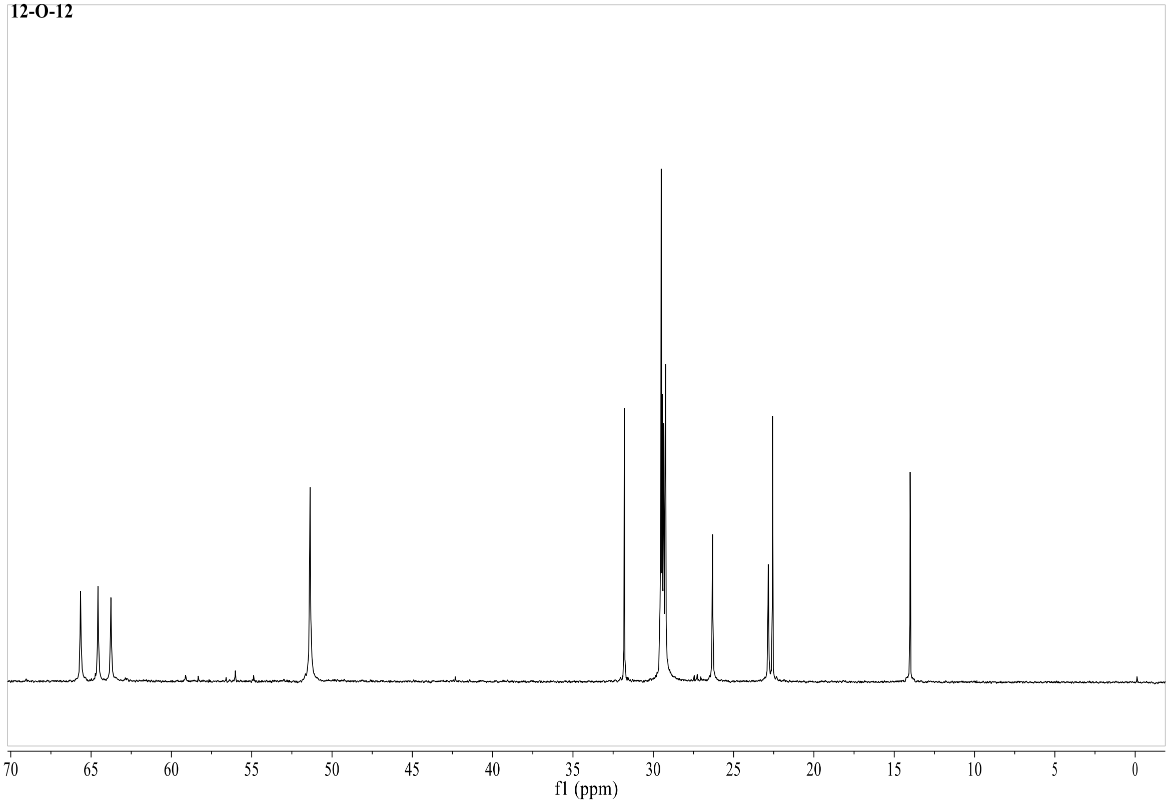

Compound **14-O-14**

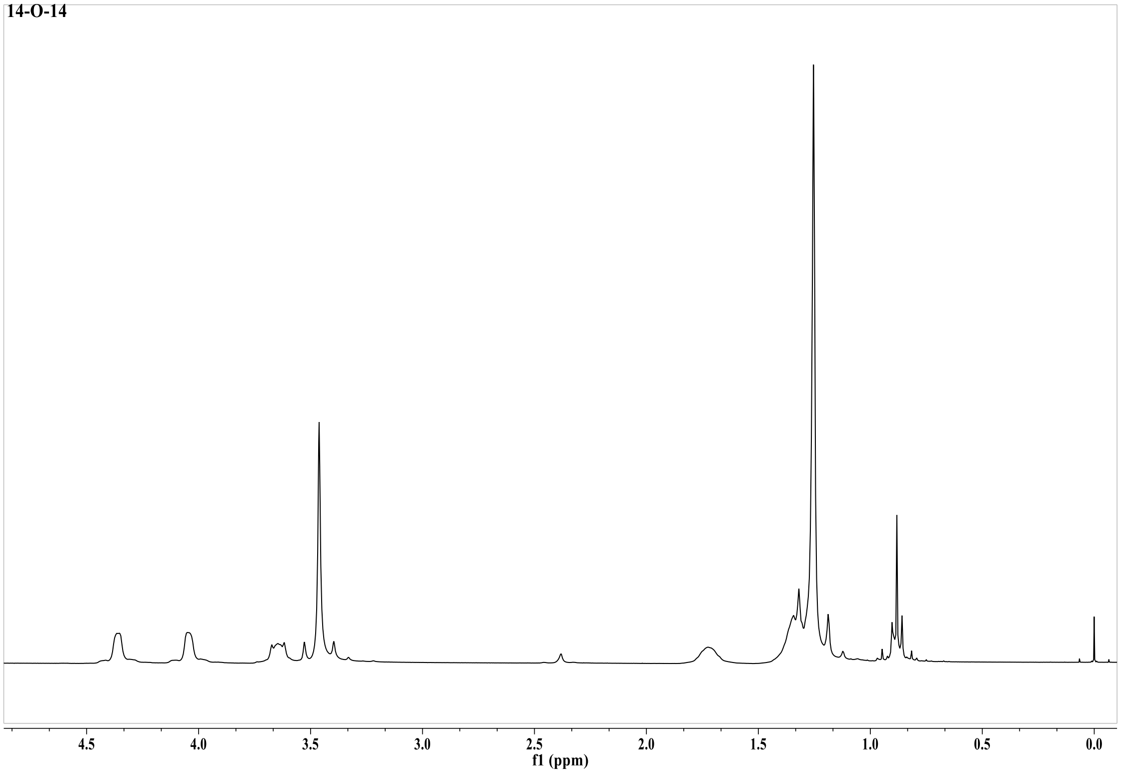

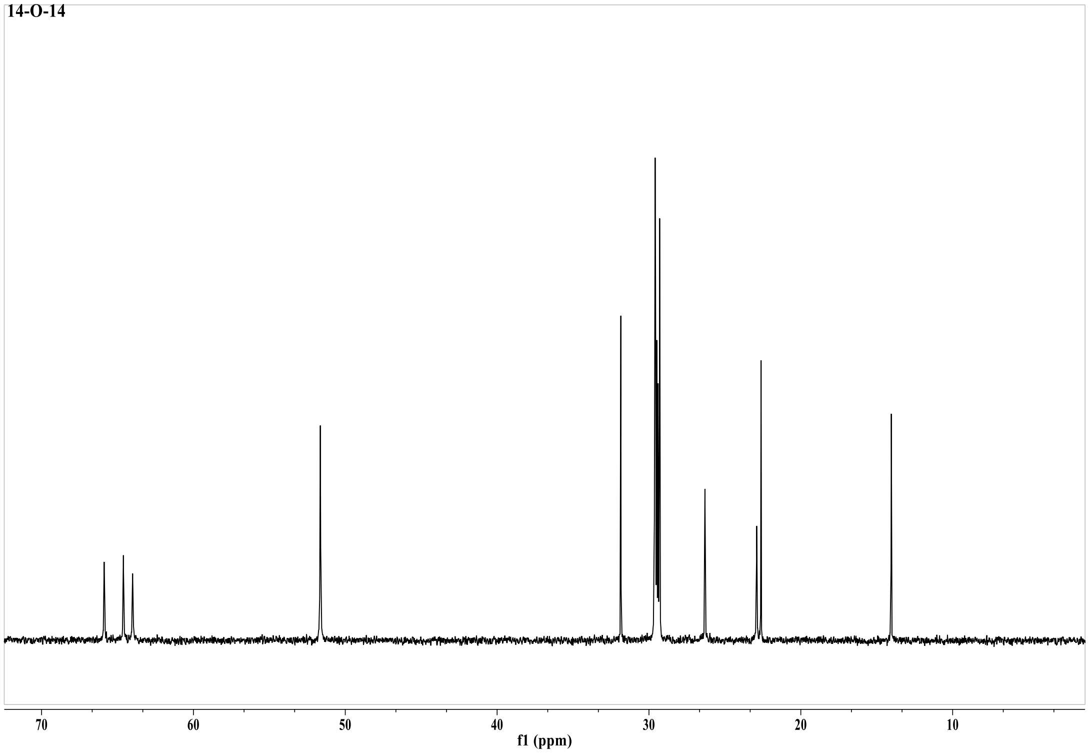

Compound **16-O-16**

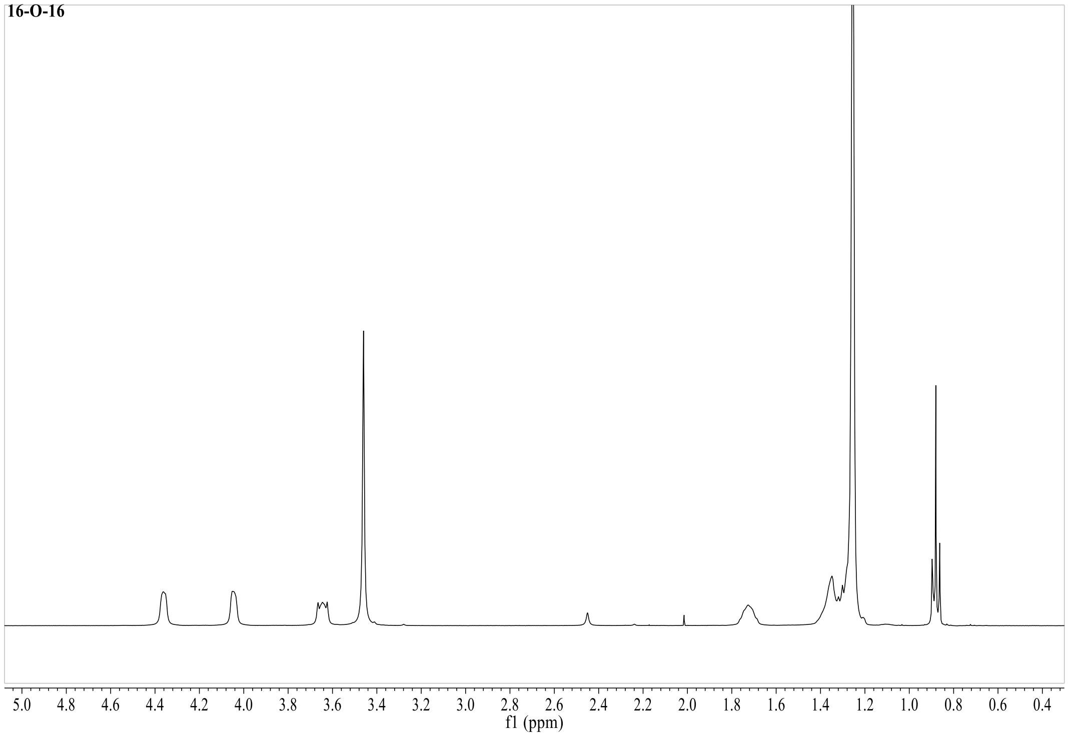

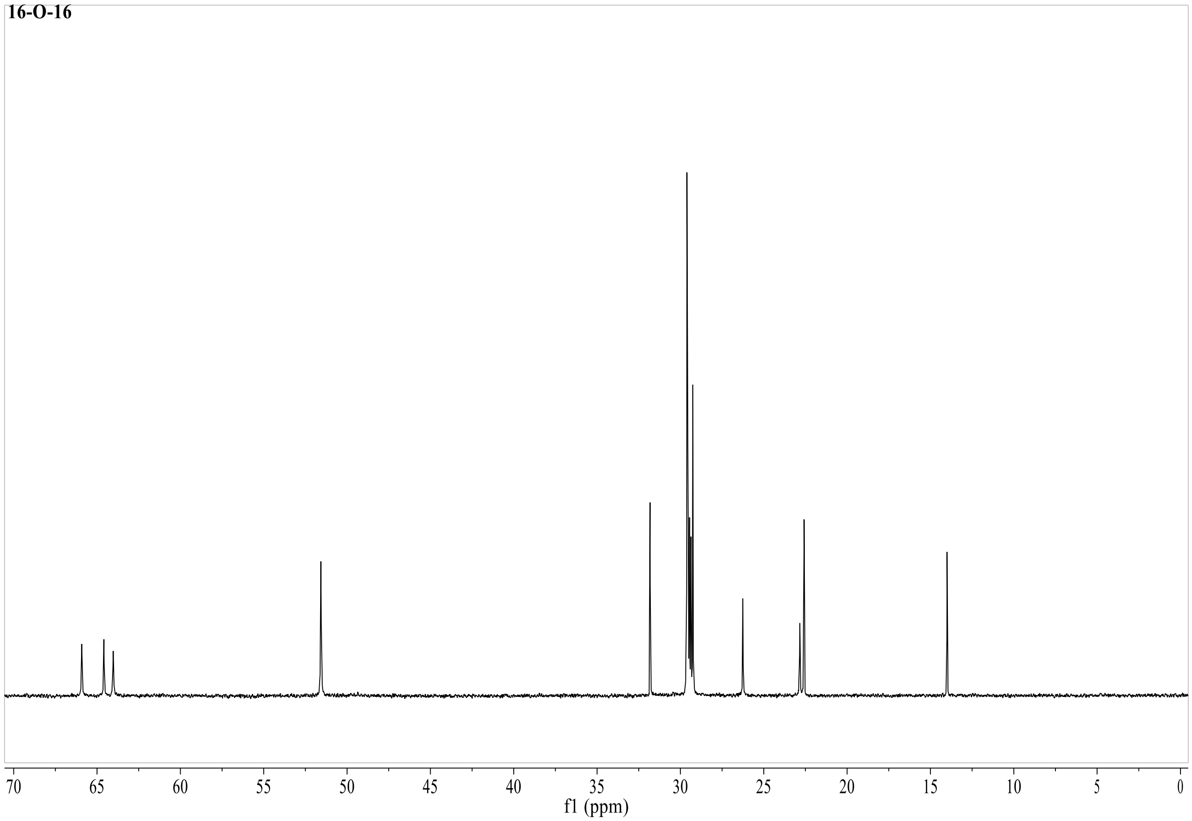

Compound **18-O-18**

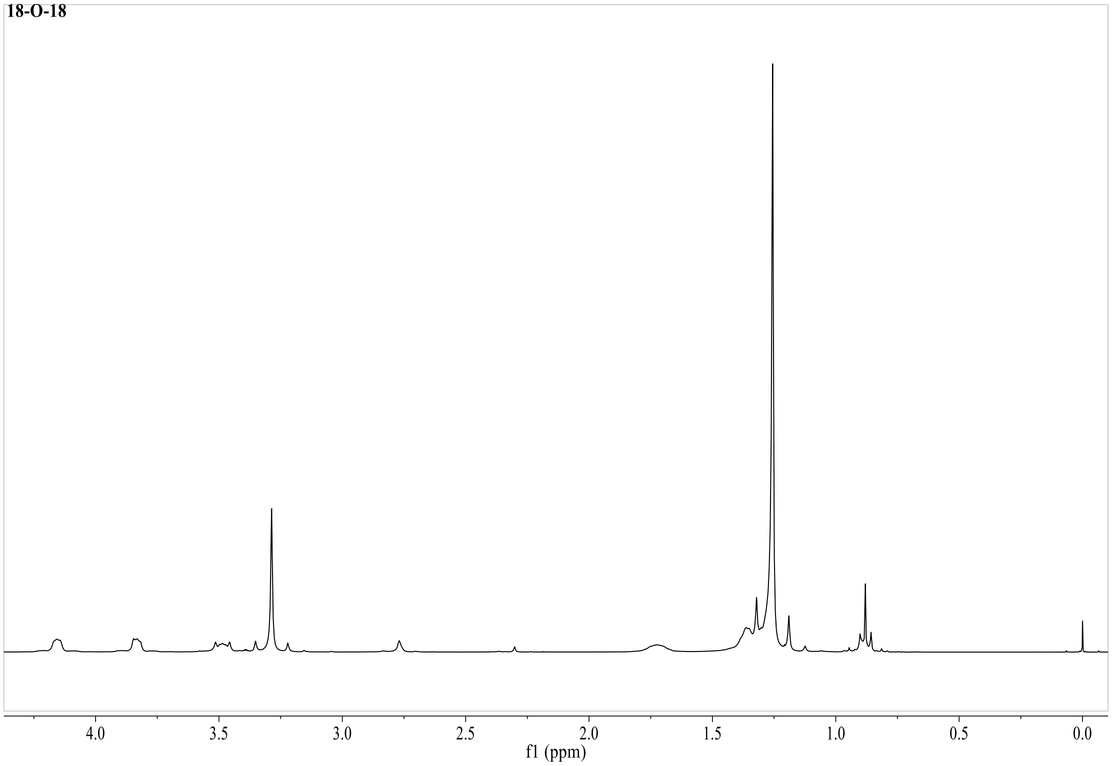

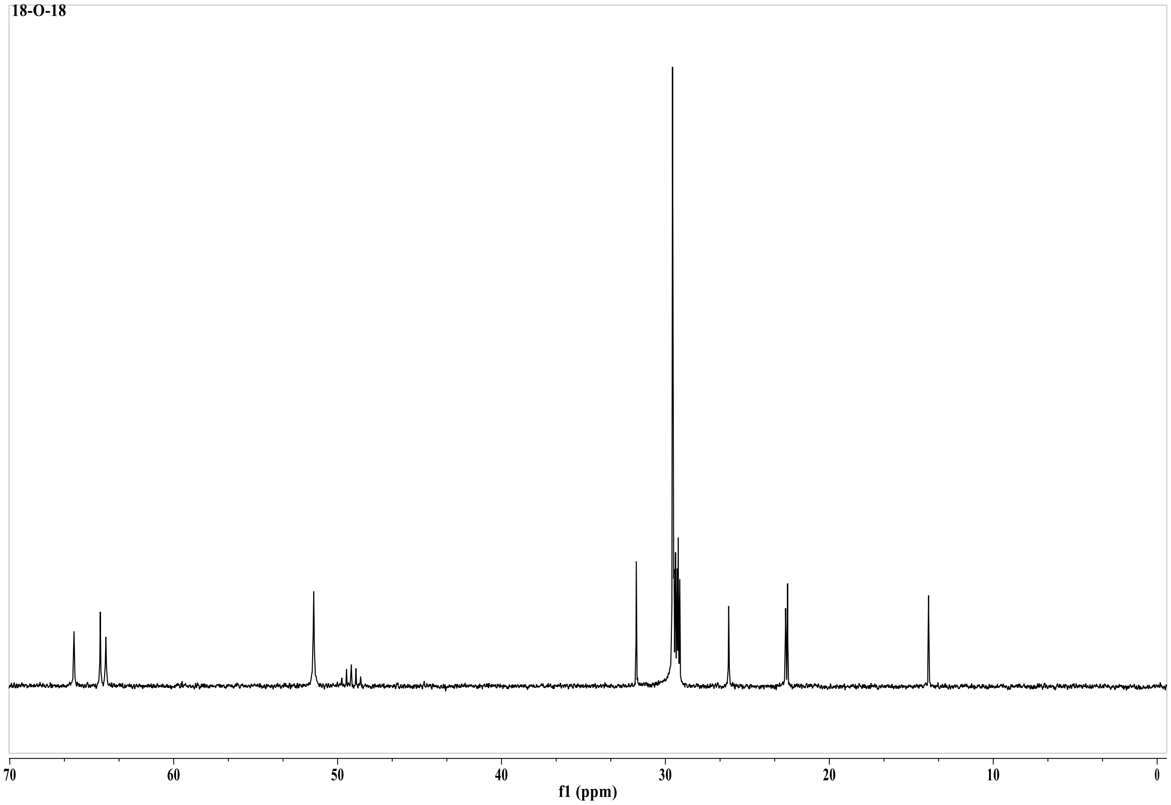

FTIR spectra

Due to the fact, that increasing the length of the alkyl substituent does not affect the shape of the FTIR spectrum, we present comparison of spectra of compound 12-O-12 (blue one) and 18-O-18 (red one).

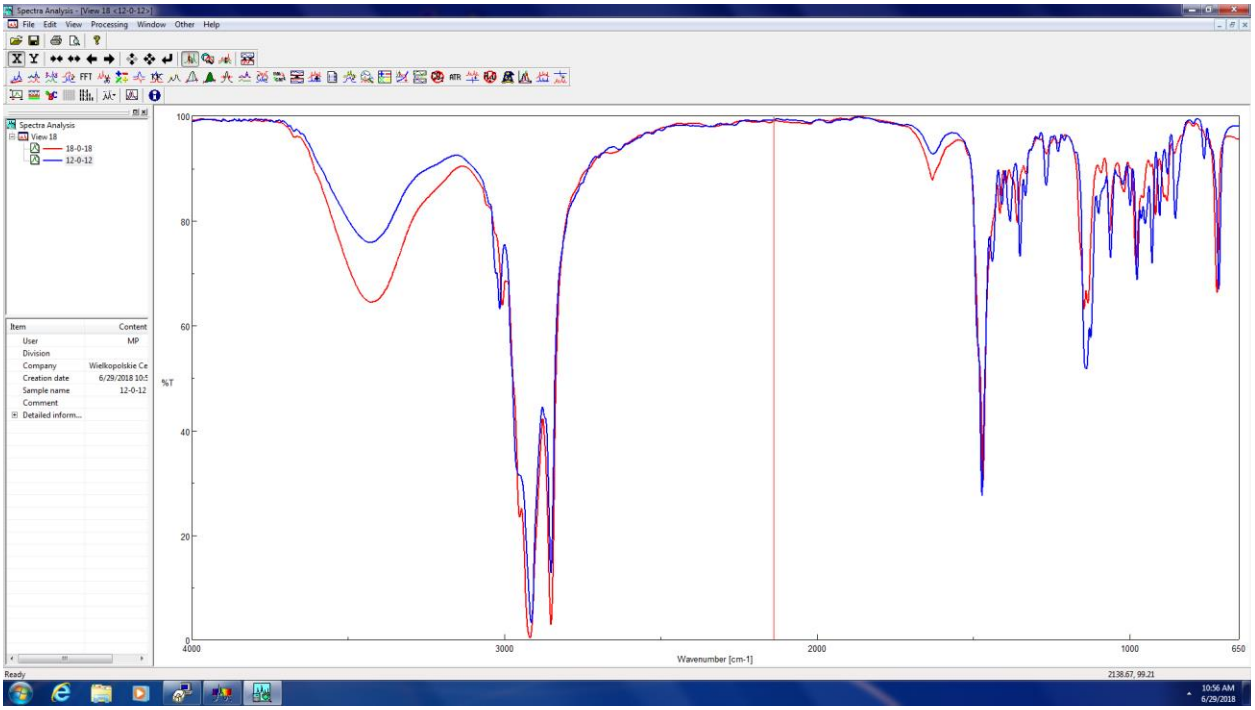

Supplement: Supplementary file 1 [file molecules-26-05759-s001.zip › molecules-1385273-supplementary.pdf]
